# Analysis of Mesostructure Characteristics and Clogging Mechanism of Pervious Concrete Based on CT Scanning and Image Processing Techniques

**DOI:** 10.3390/ma18061189

**Published:** 2025-03-07

**Authors:** Lan Liu, Taidong Guo, Zhi Cheng, Zhongzhen Wang, Xiaozhi Cheng, Zhijun Cheng, Zhe Ma

**Affiliations:** 1 School of Environment and Safety Engineering, North University of China, Taiyuan 030051, China; 20092361@nuc.edu.cn (L.L.); taidongguo@163.com (T.G.); wangzhongzhen_@163.com (Z.W.); 15275259017@163.com (X.C.); zhijuncheng@nuc.edu.cn (Z.C.); 2 China Power Construction Group Northwest Survey Design and Research Institute Co., Ltd., Xi’an 710065, China; mz605810831@163.com

**Keywords:** pervious concrete, mesostructure prediction, clog, CT scanning and image processing technique

## Abstract

This study utilized CT scanning and image processing techniques to extract and analyze the internal mesostructure and cement paste distribution of porous concrete. The effects of the mesostructure and cement paste distribution on the compressive strength and permeability were studied. Additionally, the research explored the blockage mechanisms and morphology in porous concrete, with CT scanning used to map the distribution of blockages within the material. The results indicate that the impact of the aggregate particle size on the compressive strength is much less significant than the effect of porosity. The images clearly show that the pore size is positively correlated with both porosity and aggregate size. Additionally, the distributions of pore size and cement paste thickness can be described using a lognormal distribution function and a two-parameter Weibull function, respectively. Blockage analysis revealed that the blockages were primarily concentrated within the top 0–30 mm of the porous concrete surface. As the pore size increases, the blockage depth increases, and blockages in the 10–30 mm range are challenging to remove with high-pressure water jets. A degradation model for the permeability performance of aggregate porous concrete, considering blockage consolidation, was established using parameters such as the blockage accumulation per unit area, aggregate particle size, and concrete porosity. This model provides theoretical and data-based references for evaluating the service life of porous concrete.

## 1. Introduction

It is well known that modern urban surfaces are predominantly covered by impervious pavements. Compared to natural soil, conventional concrete pavements lack the ability to breathe and permeate rainwater, leading to a series of environmental issues, such as the urban heat island effect, difficulties in flood control, and stormwater runoff pollution [1]. To address the growing challenges of climate change and urban resilience, particularly in the context of the increasing frequency and intensity of heavy rainfall events, the concept of sponge cities has gained significant attention worldwide. Sponge cities aim to enhance urban water management by integrating natural and engineered systems to absorb, store, and purify rainwater, thereby reducing flood risk and improving water resource sustainability. This approach aligns with global efforts to promote sustainable urban development and climate adaptation strategies. In recent years, many countries have adopted similar frameworks to address urban water management challenges, reflecting a broader shift toward sustainable and resilient urban planning. For instance, cities in Europe, North America, and Asia have implemented green infrastructure projects that emphasize nature-based solutions to mitigate the impacts of climate change. These initiatives highlight the importance of innovation-driven development, environmental sustainability, and the integration of technological advancements in urban planning. The development of sponge cities is not only a response to local environmental challenges but also part of a global movement toward achieving carbon neutrality and enhancing urban resilience. By focusing on sustainable urban water management, cities worldwide can better adapt to the uncertainties posed by climate change while improving the quality of life for their residents. This paper explores the principles and practices of sponge cities within a global context, aiming to contribute to the ongoing discourse on sustainable urban development and climate resilience.

Permeable concrete, a key material in sponge city construction, can effectively reduce the burden on urban drainage systems, prevent localized flooding, improve rainwater harvesting, and reduce stormwater runoff pollution [2].

The macroscopic performance of permeable concrete is determined by the physical and mechanical properties of its aggregates, cement paste, and pore structure. The strength of permeable concrete is influenced by factors such as the mixing ratio, aggregate properties, cement paste structure, and porosity, while its permeability depends on the interconnected pores within the concrete [3,4,5,6,7,8]. The skeletal structure of pervious concrete can be characterized by the number of contact bands, width of the contact bands, and thickness of the cement paste between adjacent aggregates [4]. An increase in porosity inevitably leads to an increase in permeability, which, in turn, results in a decrease in the density of the concrete. However, an increase in the permeability coefficient can reduce the compressive and flexural strengths of pervious concrete pavement mixtures [5]. The amount of fine aggregate significantly affects the permeability coefficient of the concrete mixture. It also influences the compressive strength, especially in mixtures with 10% fine aggregates, which balance both the mechanical and hydraulic properties of the mix. A water-to-binder (w/b) ratio of 0.35 results in a well-balanced pervious concrete mixture with adequate functionality and balanced mechanical and permeability performance [6]. Furthermore, studies have shown that the compressive strength of paving bricks decreases as the aggregate-to-cement ratio increases [7]. The aggregate size also has a significant impact on the freeze-thaw durability and mechanical strength of pervious concrete. Smaller aggregate sizes tend to enhance the performance of pervious concrete, while aggregates with sizes ranging from 4.75 to 9.5 mm provide excellent freeze-thaw durability. The influence of the water-to-cement ratio on pervious concrete performance is less significant compared to the effects of aggregate size and porosity. Therefore, an optimal mix design is required to strike a balance between permeability, mechanical strength, and freeze-thaw durability [8].

Therefore, understanding the relationship between the macroscopic performance, microstructure, and design factors of permeable concrete is crucial for optimizing and improving its performance. Numerous studies have investigated the impact of aggregate characteristics on the macroscopic performance of concrete. In particular, the effect of aggregate gradation on permeable concrete has been a topic of significant interest [9]. Chockalingam et al. [10] examined the effect of aggregate particle size on the strength of permeable concrete and found that increasing the aggregate size reduces compressive strength, tensile strength, and flexural strength. Cosic et al. [11] found that variations in aggregate size and type affect the bending strength of permeable concrete, while the connectivity of pores is more influenced by aggregate type. Zhang et al. [12] showed that the strength of permeable concrete with recycled aggregates decreases with an increasing aggregate crushing index. While these studies focused on the effects of aggregate type and size on the functionality of permeable concrete, they often overlooked internal factors, such as pore size and paste thickness, which also affect macroscopic performance.

Many studies have used 2D CT imaging to extract the porosity distribution of permeable concrete. The common approach involves slicing the permeable concrete into segments and extracting images [13,14,15,16,17]; the initial conditions of each case studied are shown in Table 1. However, this method is not only complex but also inadequate for capturing the full macro-permeability performance, which is influenced by complex microstructural features beyond porosity. Consequently, researchers have begun to focus on the detailed pore structure characteristics of permeable concrete [18,19,20,21]. Nonetheless, due to the limitations of 2D CT imaging and image analysis techniques, there has been less attention to pore size and pore tortuosity, which directly affect permeability. For instance, a single 2D slice cannot be used to assess pore connectivity. This approach can only achieve one-dimensional pore structure extraction and is insufficient for predicting and designing pore structures. Therefore, extracting the 3D structure of permeable concrete is crucial. Additionally, the cement paste surrounding the aggregates significantly impacts the strength and permeability of the concrete. Variations in the cement paste thickness under different aggregate sizes and characteristics affect the internal porosity of permeable concrete. With high aggregate packing densities, less cement paste is needed to fill the remaining voids. Current research on cement paste thickness remains at the observational stage, and assessing cement paste thickness in permeable concrete is challenging.

A long-standing issue with permeable concrete in service is the degradation of permeability caused by blockage from sand, clay, and other materials. In northern China, which is characterized by infrequent rain but frequent wind and dust, the impact of blockages is relatively severe [22,23,24,25]. Kayhanian et al. [26] found that most blockages consist of particles larger than 38 µm. Kia et al. [27] reviewed blockage mechanisms and maintenance methods, qualitatively summarizing the relationship between pore structure and flow particle characteristics. They suggested that regular vacuuming and pressure washing can maintain good permeability, speculating that blockages are difficult to remove due to the presence of numerous tortuous permeable channels. Deo et al. [28] investigated the impact of blockage types on blockage formation and found that coarse sand particles did not cause blockages because they could not enter smaller permeable pores. While these findings are not always consistent, they indicate that finer particles are more likely to cause blockages in permeable concrete. Several scholars [29,30,31] have tested the recovery of permeability using methods such as sweeping, pressure washing, and vacuuming. The results showed that vacuum cleaning was the most effective method for restoring permeability, followed by pressure washing, although combining both methods did not significantly improve the recovery rate. Existing research has mostly focused on quantifying the impact of blockage type and particle size on permeability, overlooking the accelerated blockage caused by blockage consolidation and the effect of the concrete’s internal pore structure on blockage development and maintenance. Moreover, there is a lack of quantitative studies on pore characteristics and blockage distribution under different blockage states (unblocked, fully blocked, and post-maintenance), leading to an unclear understanding of the blockage mechanisms in permeable concrete.

To elucidate the impact of the internal pore structure and cement paste thickness distribution on the macroscopic performance of permeable concrete, and to clarify the blockage mechanisms and maintenance methods, this study tests the compressive strength and permeability coefficient of permeable concrete with different aggregate sizes and porosities. Using medical and industrial CT scanning, the pore structure and cement paste distribution within the permeable concrete are obtained and reconstructed in 3D. The MIMICS program is used to extract the pore size distribution and cement paste thickness and to establish distribution functions. Finally, a quantitative study of the blockage distribution, depth, and pore size changes is conducted. CT scanning and image processing techniques are employed to reveal the degradation and maintenance mechanisms of permeability performance from a mesoscopic perspective, leading to the development of a permeability degradation model for permeable concrete. This study demonstrates the predictive capability of CT imaging technology for the macroscopic performance of permeable concrete and provides new insights into the comprehensive microstructural analysis of its performance.

## 2. Materials and Methods

### 2.1. Materials

The cement used in this paper is 42.5 Ordinary Portland Cement, and its main chemical composition is presented in Table 2. The physical and mechanical properties of the cement are shown in Table 3. The aggregate used in this study was natural basalt with a particle size of 5–10 mm. The aggregate was washed with water and dried at room temperature. Its properties were tested according to the JGJ52-2006 standard [32]. The physical and mechanical properties of the aggregates are shown in Table 4.

**Figure 1:**
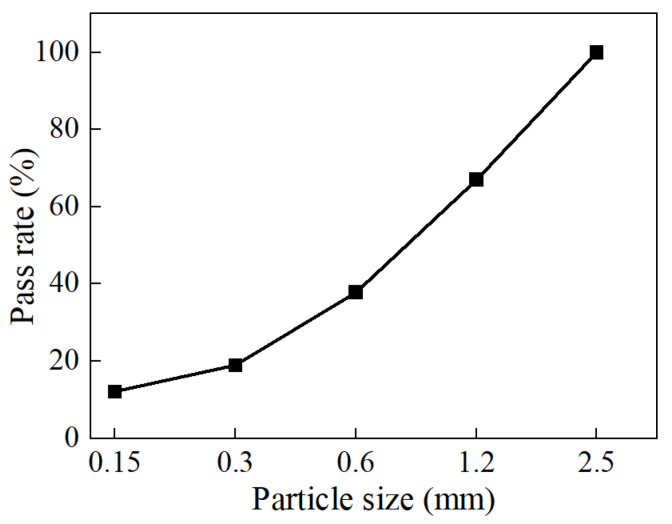
Particle grading of contaminants.

The clogging material used in this study was sourced from planting soil in road green belts and was primarily composed of clay and sand. Before the experiment, the clogging material was dried. Considering that large particles do not significantly contribute to clogging, particles larger than 2.5 mm were removed by sieving. The appearance and grading of the clogging material are shown in Figure 1.

### 2.2. Mix Proportions

To investigate the pore characteristics and clogging mechanisms of pervious concrete with different aggregate sizes and porosities, 5–10 mm aggregates were divided into two size ranges using an 8 mm sieve: 5–8 mm and 8–10 mm. The design porosities were set to 18.3%, 24.2%, and 30.1%. The mix proportions of the pervious concrete were calculated using the volumetric method according to the standard CJJ/T135-2009 [33]. The mixing ratios of the samples are shown in Table 5. The specimen dimensions were 100 mm × 100 mm × 100 mm, and all specimens were cured in water at room temperature for 28 days.

### 2.3. Test Methods

#### 2.3.1. Compressive Strength and Permeability Coefficient Testing

**Figure 2:**
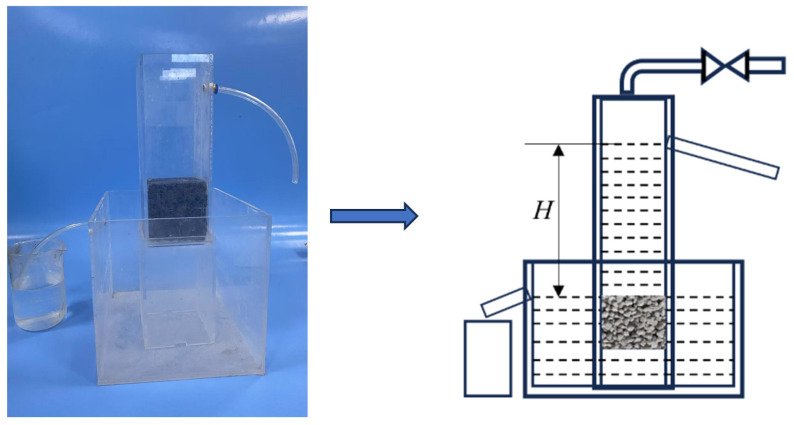
Water Permeability Testing Device.

The 28-day compressive strength of the pervious concrete was tested according to the Chinese standard GB/T 50081-2002 [34]. The permeability coefficient was determined according to the Chinese standard CJJ/T 135-2009 [33], and three samples were set for each group. The test setup is shown in Figure 2. The specimen size was φ100 mm × 50 mm, and three samples were prepared for each group. To measure the diameter and thickness of the cylindrical specimens, a steel straightedge was used. The average of two separate measurements for each dimension was then calculated to determine the upper surface area (A) of the specimen.

Before starting the measurements, the perimeters of each specimen were sealed with a waterproof tape. After curing, the specimen was placed in a vacuum device and subjected to a vacuum pressure of (90 ± 1) kPa for 30 min. Simultaneously, water was added to cover the specimen, and the water level was maintained 100 mm above the specimen. Once the vacuum was stopped, the specimen was soaked for 20 min.

Next, the specimen was removed and placed into the permeability test device, where the specimen and the permeability cylinder were connected, and the overflow was sealed into a tank. The valve was then opened, allowing the water to flow into the container until it overflowed. After the overflow stabilized at both the sink and the permeable cylinder, a measuring cylinder was used to collect water from the outlet. The volume of water flowing out over a 5-min period (Q) was recorded. This measurement was repeated three times, and the average value was calculated.

Finally, the difference in water levels between the permeable cylinder and the overflow tank (H) was measured using a steel ruler, and the temperature of the water in the overflow tank (T) was recorded using a thermometer. The permeability coefficient was then calculated using Equation (1).

(1) $$K_{T}=\frac{Qh}{SHt}$$

where *K_T_* is the permeability coefficient (mm/s) of the specimen at water temperature *T* °C, *Q* is the volume of water seeping out in a given time (mm^3^), h is the thickness of the specimen (mm), *S* is the surface area of the specimen’s upper surface (mm^2^), and *H* is the water level difference (mm).

#### 2.3.2. Clogging Testing

The clogging simulation device for pervious concrete is the same as the permeability coefficient testing device shown in Figure 2. First, the initial permeability coefficient of each specimen before clogging was measured using the constant-head method with a water head height of 100 mm and a test duration of 1 min. Then, 5 g of the clogging material was evenly sprinkled on the surface of the specimen. Water was added to a height of 100 mm, and the permeability coefficient after adding the clogging material was tested while maintaining a constant water head height and continuously stirring to ensure that the clogging material penetrated the internal pores of the specimen. The water flow over 5 min was recorded, and the permeability coefficient was then calculated. The specimen was then removed from the testing device and dried. This step simulates the consolidation and hardening process of clay in pervious concrete pavements after rainfall. The process was repeated until the permeability coefficient decreased to 0.2 mm/s, at which point the aggregate-pervious concrete was considered to be fully clogged. Notably, the “Technical Specification for Pervious Cement Concrete Pavement” requires an initial permeability coefficient greater than 0.5 mm/s; therefore, it is reasonable to consider the specimen fully clogged when the permeability coefficient drops to 0.2 mm/s. Once the specimen was fully clogged, it was placed in the testing device, and the loose clogging material on the surface was removed using a vacuum cleaner. The surface of the specimen was then cleaned vertically using a high-pressure water jet. Based on the pressure values of actual cleaning vehicles and the literature, the water jet pressure was set to 10 MPa with a cleaning duration of 30 s [32,33,34,35]. After cleaning the clogging material, the permeability coefficient was retested. Each group consisted of three specimens, and the final result was the average of the test results for each group.

#### 2.3.3. Methods of Mesoscopic Analysis

**Figure 3:**
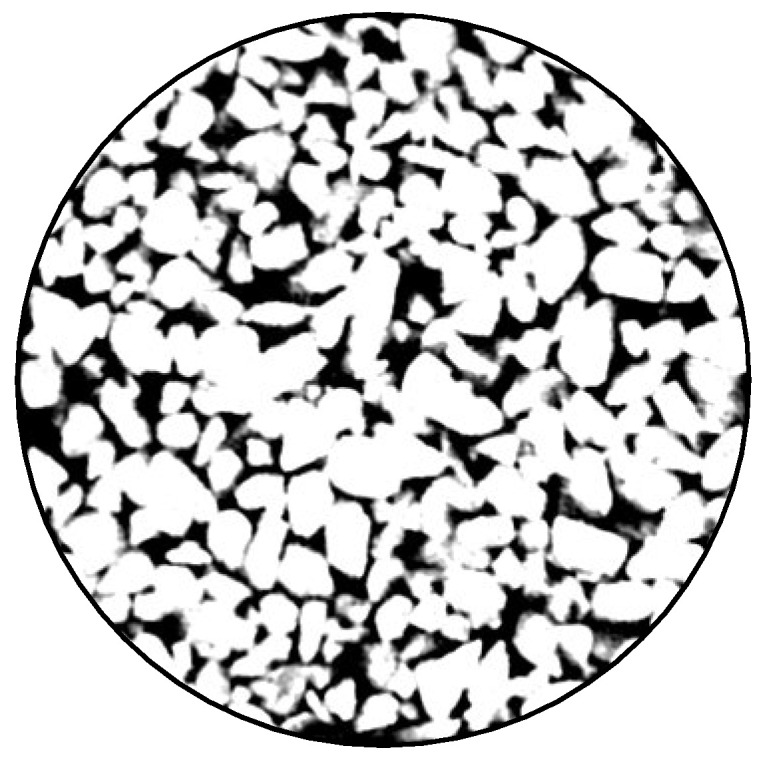
CT scanned specimens.

Mesoscopic analysis was performed using CT scanning technology with MIMICS MEDICAL 17.0 software. First, a small cylindrical specimen measuring *Φ* 30 mm × 30 mm was drilled from the center of a larger specimen measuring 100 mm × 100 mm × 100 mm. The internal pore structure of the specimen was scanned using a medical CY scanner (model Discovery CT750 HD, GE HealthCare, Chicago, IL, USA, 512 × 512 pixels). Due to the different X-ray absorption rates of the components within the pervious concrete, internal interface images were generated. The resulting ImageJ format images are shown in Figure 3.

**Figure 4:**
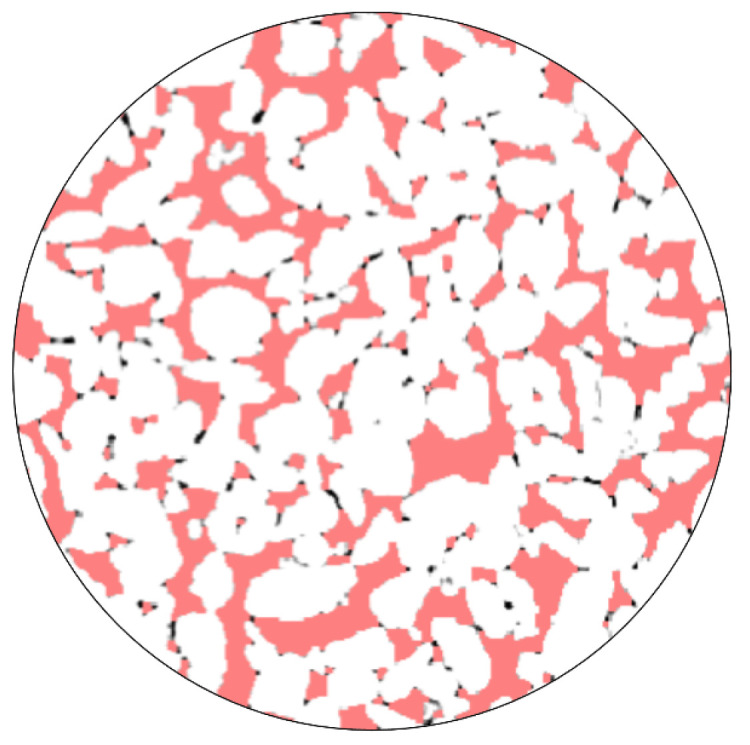
Extraction of pore distribution.

**Figure 5:**
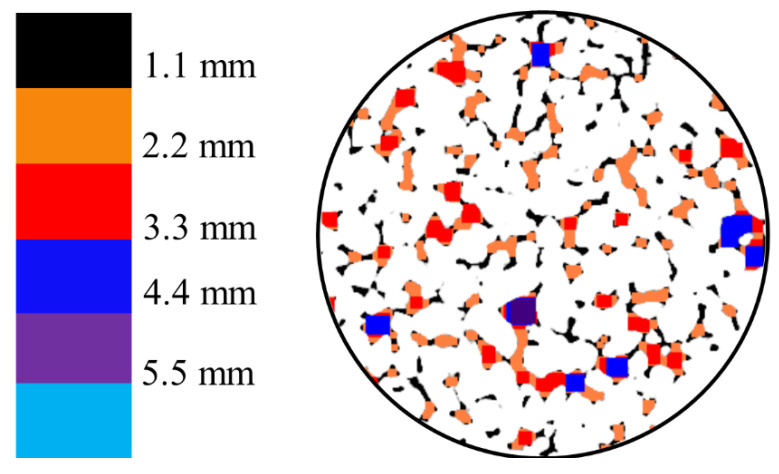
The color differentiation of pores with varying diameters.

**Figure 6:**
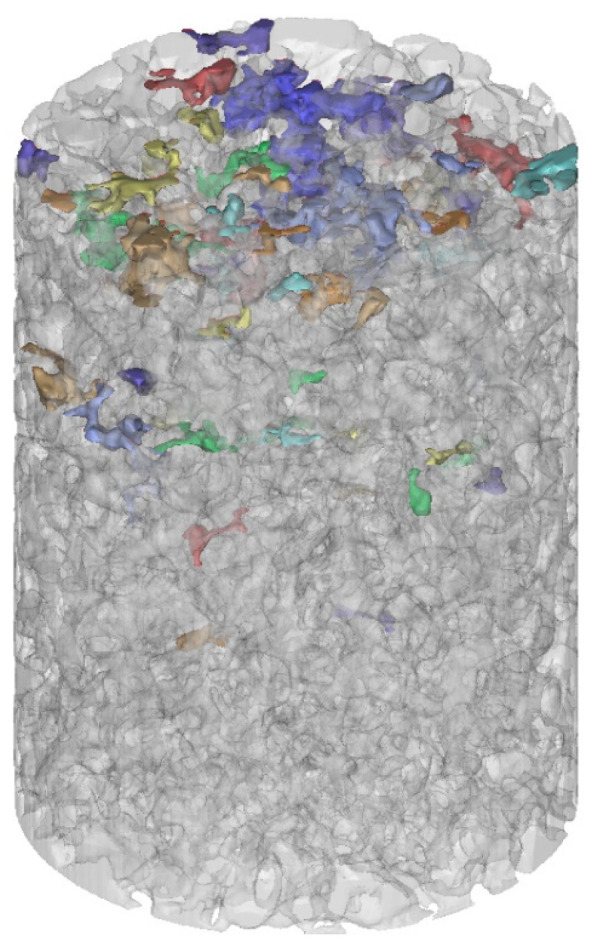
3D reconstruction of the pervious concrete.

Using the sample images obtained from Figure 3, threshold segmentation was performed in the MIMICS software based on the grayscale values to extract the pore distribution, as shown in Figure 4. Additionally, morphological operations (opening operation) were applied. The specimen was divided into pixels of 0.195 mm with a resolution of 512 × 512 pixels. The pore distribution was identified by grayscale value recognition and differentiation of colored pixels. Figure 5 illustrates a schematic representation of this process. In this study, 80 images were scanned across three sections. Subsequently, the 3D reconstruction functionality of MIMICS was utilized to obtain the three-dimensional pore structure of the pervious concrete, as shown in Figure 6.

**Figure 7:**
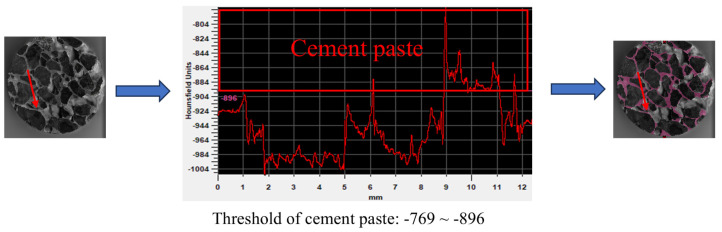
Extraction of the cement paste thickness distribution.

Due to the low and thinly distributed cement matrix content in pervious concrete, which has a density close to that of the aggregates, it is challenging to distinguish the cement matrix from the aggregates using a lower-resolution medical CT scanner [36]. Therefore, a high-resolution industrial CT scanner was selected (model Discovery MicroXCT-400, pixel size of 37 μm). The method for extracting the cement paste is illustrated in Figure 7. First, random lines were drawn on the CT scan images of the pervious concrete using MIMICS software to extract the pixel grayscale values along these lines. These grayscale values were then used to differentiate the aggregates, pores, and cement matrix. Additionally, the images were divided into horizontal line segments, and the lengths of all the line segments were extracted and recorded. The length of these line segments represents the thickness of the cement paste, allowing for the determination of a distribution pattern. Three specimens were extracted and averaged for each group.

## 3. Results and Discussion

### 3.1. Compressive Strength and Permeability Coefficient

**Figure 8:**
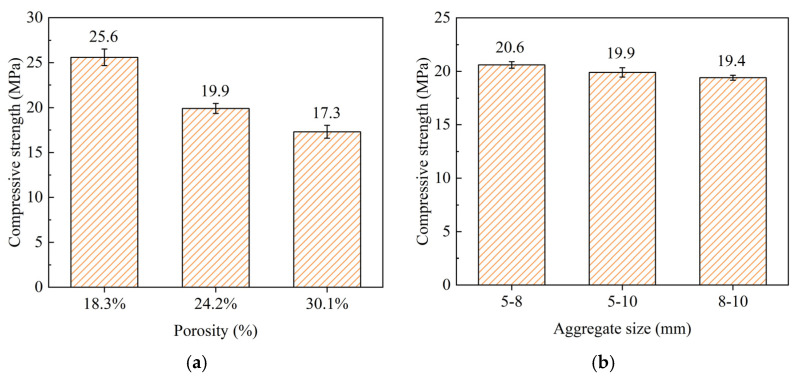
Compressive strength: (**a**) different porosities and (**b**) different aggregate sizes.

Figure 8a shows the compressive strength of pervious concrete with different porosities, where the aggregate size ranges from 5–10 mm. The data indicate that as the porosity increased, the compressive strength decreased. Specifically, the compressive strength was 25.6 MPa at a porosity of 18.3%, 19.9 MPa at a porosity of 24.2%, and 17.3 MPa at a porosity of 30.1%. This trend demonstrates an inverse relationship between porosity and compressive strength in pervious concrete. As porosity increased, the volume of voids within the concrete structure increased, leading to a reduction in load-bearing capacity and, consequently, lower compressive strength. Figure 8b presents the compressive strength of pervious concrete with a constant porosity of 24.2% but varying aggregate sizes. The compressive strengths for aggregate sizes of 5–8 mm, 5–10 mm, and 8–10 mm were 20.6 MPa, 19.9 MPa, and 19.4 MPa, respectively. The results suggest that smaller aggregate sizes slightly enhanced the compressive strength of the pervious concrete. This is likely because smaller aggregates provide denser packing, which may reduce the void spaces within the matrix, thus improving the material’s ability to withstand compressive loads. However, the differences in compressive strength between the various aggregate sizes were not substantial, indicating that the aggregate size within the tested range had a relatively minor effect compared to porosity. The results illustrate that porosity has a more pronounced impact on the compressive strength of pervious concrete compared to aggregate size. High porosity levels lead to a significant reduction in compressive strength due to the increased void content, which compromises the structural integrity of the concrete [37]. In contrast, variations in aggregate size within the range of 5–10 mm showed only marginal differences in compressive strength, suggesting that while aggregate size can influence the packing density and, subsequently, the compressive strength, its effect is less critical than that of porosity.

**Figure 9:**
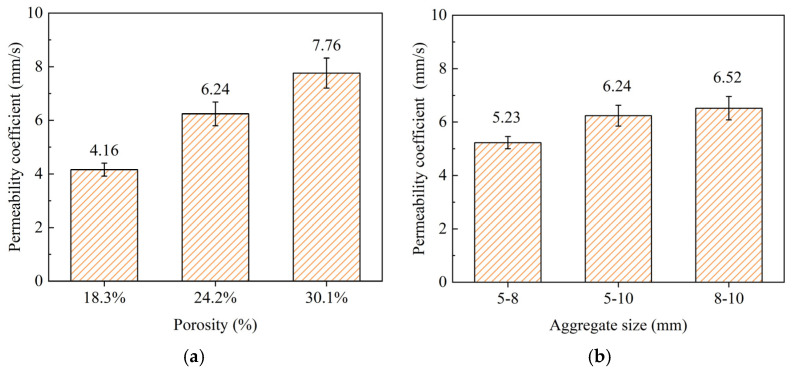
Permeability coefficient: (**a**) different porosities; (**b**) different aggregate sizes.

Figure 9a illustrates the relationship between the porosity and permeability coefficients of the permeable concrete. As expected, the permeability coefficient increased with increasing porosity levels. Specifically, permeable concrete with porosities of 18.3%, 24.2%, and 30.1% exhibited permeability coefficients of 4.16 mm/s, 6.24 mm/s, and 7.76 mm/s, respectively. This trend can be attributed to the fact that higher porosity provides more interconnected void spaces, facilitating the flow of water through the concrete. The increase in permeability with higher porosity is consistent with previous studies and highlights the importance of porosity in determining the permeability performance of permeable concrete. Figure 9b presents the permeability coefficients of permeable concrete with different aggregate sizes. The permeability coefficients for concrete prepared with aggregate sizes of 5–8 mm, 5–10 mm, and 8–10 mm were measured at 5.23 mm/s, 6.24 mm/s, and 6.52 mm/s, respectively. The results indicate that larger aggregate sizes generally lead to higher permeability, likely due to the formation of larger pores that enhance water flow. However, the difference in permeability between 5 and 10 mm and 8 and 10 mm aggregate sizes was relatively small, suggesting that beyond a certain point, further increases in aggregate size contribute less significantly to permeability. This observation suggests a potential trade-off between aggregate size and other factors, such as strength, which should be considered when optimizing the design of permeable concrete for specific applications.

### 3.2. Pore Structure and Cement Paste Distribution

#### 3.2.1. Pore Structure

**Figure 10:**
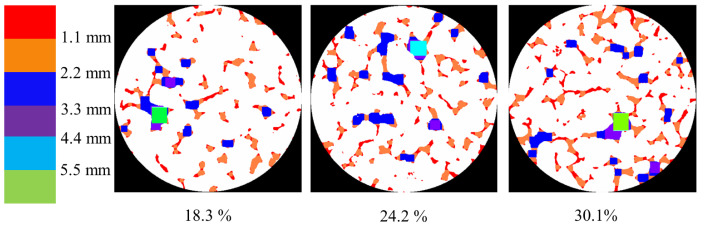
Pore distribution of the specimens with different porosities.

Figure 10 shows the pore distribution in pervious concrete with different porosities obtained through CT scan processing. It can be observed that pores smaller than 2.2 mm constitute the majority. As the porosity increased, the number of pores significantly increased, and the complexity of the pore sizes also increased. This complexity is indicative of the variability in the pore formation processes and the distribution of voids within the concrete. Higher porosity allows for a more heterogeneous pore structure, which can affect the material’s overall mechanical properties and permeability. In pervious concrete with a porosity of 30.1%, pores with diameters ranging from 2.2 mm to 4.4 mm constitute the majority.

**Figure 11:**
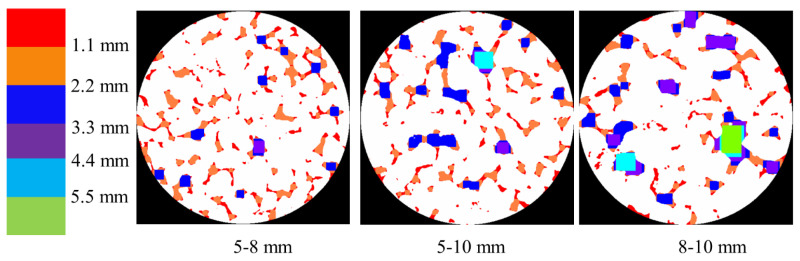
Pore distribution of specimens with different aggregate sizes.

Figure 11 shows the pore size distribution of pervious concrete with a porosity of 24.2% and three different aggregate sizes. It can be observed that as the aggregate size increased, the proportion of larger pores also increased. For pervious concrete with an aggregate size of 5–8 mm, the pores were predominantly smaller than 2.2 mm in size. In pervious concrete with a 5–10 mm aggregate size, there was a noticeable increase in pores with diameters between 2.2 mm and 3.3 mm. When the aggregate size was increased to 8–10 mm, the number of small pores less than 2.2 mm significantly decreased, with the majority of pores falling within the 3.3 mm to 4.4 mm range. Larger aggregate sizes tend to increase the proportion of larger pores, which can negatively impact the compressive strength and structural integrity of pervious concrete. Larger voids can create weak points in concrete, potentially reducing its load-bearing capacity. The increase in the number of larger pores with larger aggregate sizes generally enhanced the permeability of the pervious concrete. This characteristic is advantageous for applications requiring high drainage capacity but may be less desirable for structural applications needing higher strength.

**Figure 12:**
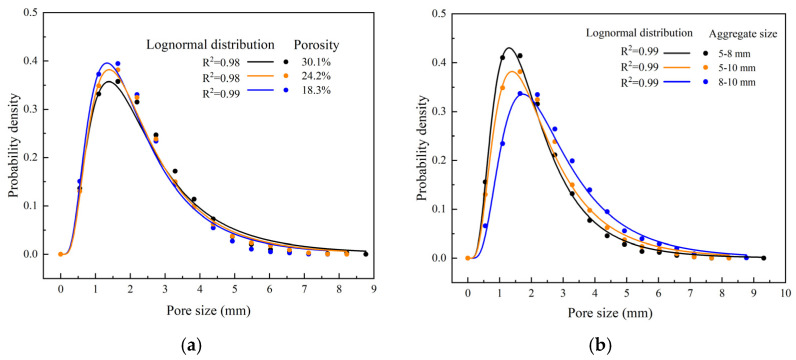
Probability density distribution of pore sizes: (**a**) different porosities and (**b**) different aggregate sizes.

Figure 12 illustrates the probability density distribution of pore sizes calculated from the planar CT images shown in Figure 10 and Figure 11 (three cross-sections, totaling 80 images of each specimen, each group containing three specimens). Interestingly, the distribution of pore sizes can be described by a lognormal distribution, as shown in Equation (2) and Table 6. In this equation, parameters *μ* and *σ* represent the mean and standard deviation of the logarithm of the variable, respectively.

(2) $$f\left(x,\sigma ,\mu \right)=\frac{1}{x\sigma \sqrt{2\pi }}\exp\left[-{\left(\ln x-\mu \right)}^{2}/2\sigma^{2}\right]$$

For a porosity of 30.1%, the mean pore size (on a logarithmic scale) is the highest at *μ* = 2.62 with a standard deviation of *σ* = 1.90. This indicates a wide distribution of pore sizes, with a tendency toward larger pores. At a porosity of 24.2%, the mean pore size decreases to *μ* = 2.39, and the distribution becomes slightly narrower with *σ* = 1.64. This suggests a higher concentration of medium-sized pores compared to the 30.1% porosity level. For the lowest porosity of 18.3%, the mean pore size is *μ* = 2.48, and the standard deviation is *σ* = 1.69. This level of porosity still maintains a relatively wide distribution but with fewer large pores compared to the highest porosity.

For aggregate sizes of 5–8 mm, the mean pore size is *μ* = 2.22, with a standard deviation of *σ* = 1.45. This indicates the predominance of smaller pores due to the smaller aggregate size. When the aggregate size increased to 5–10 mm, the mean pore size increased significantly to *μ* = 2.87, with a standard deviation of *σ* = 1.81. This reflects the substantial presence of larger pores. For aggregate sizes of 8–10 mm, the mean pore size is *μ* = 2.48, and the standard deviation is *σ* = 1.69, similar to the 18.3% porosity distribution. This suggests that larger aggregate sizes contribute to a moderate increase in pore size, maintaining a balance between small and large pores.

The pore size distribution in pervious concrete, as revealed by CT scanning, can be effectively modeled using a lognormal distribution. The analysis shows that higher porosities lead to a broader range of pore sizes, with a greater presence of larger pores. Similarly, an increase in the aggregate size results in larger average pore sizes and a wider distribution. These insights are crucial for optimizing the mix design of pervious concrete to achieve the desired permeability and mechanical properties.

#### 3.2.2. Cement Paste Distribution

**Figure 13:**
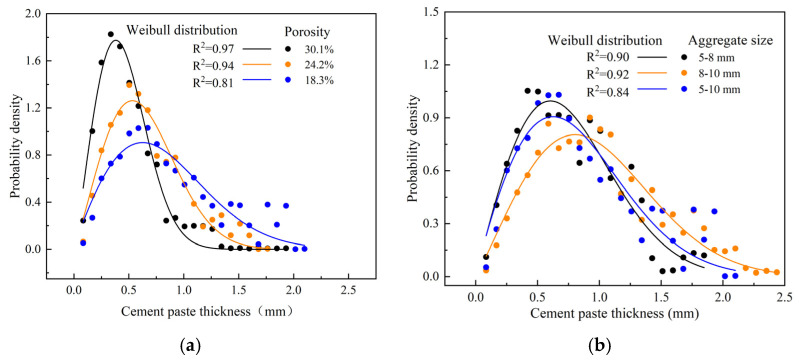
Probability density distribution of aggregate sizes: (**a**) different porosities and (**b**) different aggregate sizes.

High-precision industrial CT scanning was used to obtain cross-sectional images of each specimen, from which the distribution of the cement paste thickness and proportion of cement paste overlap areas were calculated, as shown in Figure 13. Interestingly, the thickness distribution of the cement paste can be well described by a two-parameter Weibull distribution, with the probability density function given in Equation (3).

(3) $$f\left(x,\lambda ,k\right)=\frac{k}{\lambda }{\left(\frac{x}{\lambda }\right)}^{k-1}\cdot \exp\left[-{\left(\frac{x}{\lambda }\right)}^{k}\right]$$

where, the symbol λ represents the scale parameter, while the symbol *k* represents the shape parameter. Table 7 shows the proportion parameters and shape parameters of pervious concrete with respect to the Weibull distribution function.

For a porosity of 30.1%, the scale parameter *λ* = 0.51 and shape parameter *k* = 2.16 indicate a relatively thin and consistent distribution of cement paste. At a porosity of 24.2%, the scale parameter increased to *λ* = 0.71 with a similar shape parameter *k* = 2.15, suggesting a thicker distribution of cement paste with slight variations. For the lowest porosity of 18.3%, the scale parameter further increased to *λ* = 0.92 while the shape parameter decreased to *k* = 1.91, indicating a thicker and more variable distribution of cement paste.

For aggregate sizes of 5–8 mm, the scale parameter *λ* = 0.86 and shape parameter *k* = 1.99 suggest a moderately thick distribution of cement paste with some variations. For aggregate sizes of 5–8 mm, the scale parameter *λ* = 0.86 and shape parameter *k* = 1.99 suggest a moderately thick distribution of cement paste with some variations. For aggregate sizes of 8–10 mm, the scale parameter is *λ* = 0.92 with a shape parameter of *k* = 1.91, similar to the 18.3% porosity distribution, indicating a thicker and more variable distribution of cement paste.

The thickness distribution of the cement paste in pervious concrete can be effectively modeled using a two-parameter Weibull distribution. The analysis shows that higher porosities lead to a thinner and more consistent distribution of cement paste [38]. Similarly, larger aggregate sizes result in a thicker and more consistent distribution of cement paste. These insights are crucial for optimizing the mix design of pervious concrete to achieve the desired mechanical properties and durability.

Taking the two-parameter Weibull distribution of cement paste thickness as an example, we compared the lognormal distribution and Weibull distribution using Origin software (https://www.originlab.com/). The comparison data are presented in Table 8.

After comparison, the goodness-of-fit of the two-parameter Weibull distribution was slightly higher than that of the lognormal distribution. Therefore, a two-parameter Weibull distribution was selected.

**Figure 14:**
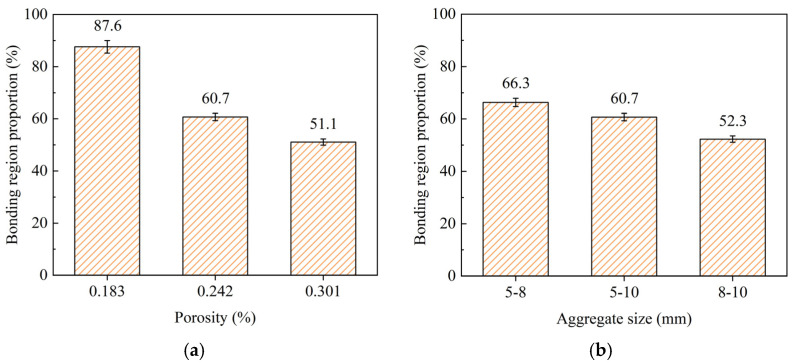
Bonding region proportion of cement paste: (**a**) different porosities and (**b**) different aggregate sizes.

Figure 14 shows the proportion of the cement paste bonding region area in the specimens with different porosities and aggregate sizes. As shown in the figure, as the porosity decreases, the proportion of the bonding region area significantly increases, leading to a notable improvement in strength. As the aggregate size decreases, the proportion of the paste bonding region increases slightly, resulting in a minor enhancement in the strength of the pervious concrete. A possible reason for this is that smaller aggregate sizes have a larger specific surface area, which increases the contact area between aggregates and consequently leads to a higher proportion of cement paste bonding region.

### 3.3. Analysis of Clogging Mechanisms

#### 3.3.1. The Deterioration Process of Permeability

**Figure 15:**
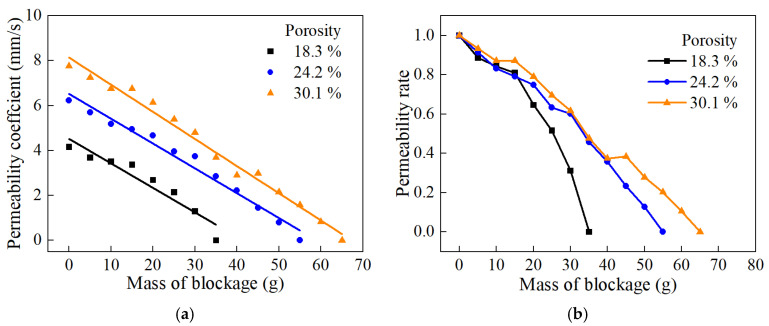
Degradation of water permeability under different porosities: (**a**) permeability coefficient; (**b**) permeability rate.

Figure 15 shows the curves of the pervious concrete with different porosities as a function of the amount of clogging material. It is evident that as the amount of clogging material increased, the pores gradually became blocked, leading to a deterioration in permeability. From Figure 15a, it can be observed that pervious concrete with a higher porosity initially has a higher permeability coefficient and can accommodate more clogging material. Figure 15b indicates that pervious concrete with a porosity of 18.3% experiences a rapid decrease in permeability with increasing amounts of clogging material, whereas the permeability of concrete with a porosity of 30.1% decreases more slowly as the amount of clogging material increases. Pervious concrete with higher porosity develops clogging more slowly, demonstrating better anti-clogging performance.

**Figure 16:**
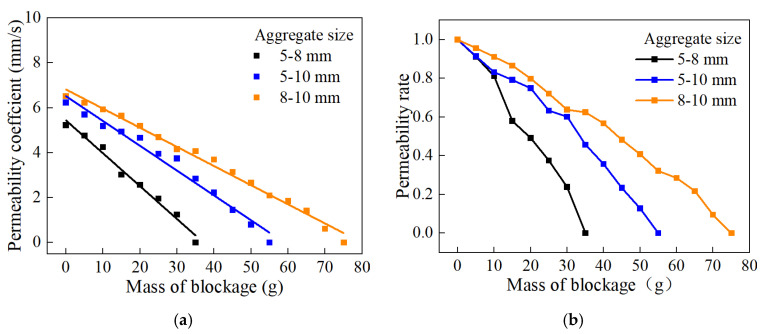
Degradation of water permeability under different aggregate sizes: (**a**) permeability coefficient; (**b**) permeability rate.

Figure 16 shows the curves of the permeability coefficient as a function of the amount of clogging material for different aggregate sizes. It can be observed from the figure that, at the same total porosity, the initial permeability coefficient is highest for specimens with 5–8 mm aggregate size, lowest for those with 8–10 mm aggregate size, and intermediate for those with 5–10 mm aggregate size. This is because specimens made with smaller aggregate sizes have smaller pore sizes and more tortuous pathways for water flow. At the same porosity, pervious concrete with 8–10 mm aggregate size can accommodate more clogging material and the clogging develops more slowly, whereas concrete with 5–8 mm aggregate size accommodates less clogging material and the clogging develops fastest, with 5–10 mm aggregate size falling in between. The possible reason is that, with the same porosity, pervious concrete made with larger aggregate sizes has larger pore sizes, allowing clogging material to penetrate deeper into the concrete under the scouring of water, thereby avoiding accumulation on the surface and resulting in slower clogging development [39]. In contrast, pervious concrete made with smaller aggregate sizes has relatively smaller pore sizes and higher tortuosity, making it difficult for clogging material to enter the specimen and causing its accumulation on the surface, leading to faster clogging development.

**Figure 17:**
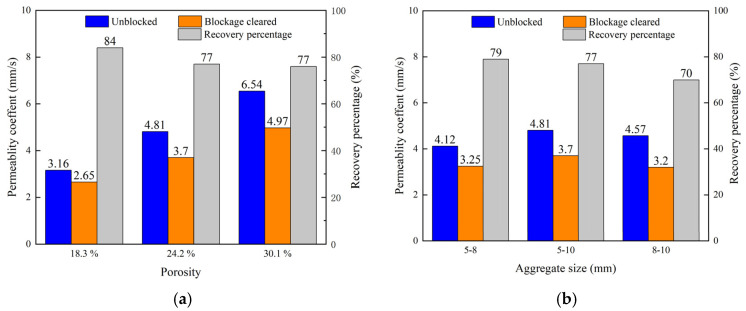
Degree of permeability recovery after cleaning: (**a**) different porosities and (**b**) different aggregate sizes.

Figure 17a shows the changes in the permeability coefficient of the pervious concrete with different porosities before and after the clogging removal. As shown in the figure, after vacuum suction and high-pressure water jet cleaning, the permeability coefficient recovery was quite high for all porosities. Pervious concrete with lower porosity showed the best recovery, likely because its smaller pore sizes caused clogging material to accumulate on the surface, making it easier to remove. Conversely, in pervious concrete with higher porosity, the clogging material penetrated deeper into the specimen, and the high-pressure water jet could not dislodge the internally consolidated clogging material, resulting in a relatively lower recovery of the permeability coefficient. Figure 17b shows the degree of recovery of the permeability coefficient for pervious concrete made with aggregates of different sizes. It can be observed that concrete made with 5–8 mm aggregate shows the highest recovery, while that made with 8–10 mm aggregate shows the lowest. The reason is similar to the aforementioned description: pervious concrete made with smaller aggregate sizes has smaller pore sizes and higher tortuosity, causing the clogging material to accumulate more on the surface, making it easier for the high-pressure water jet to dislodge it. In contrast, pervious concrete made with larger aggregate sizes has relatively larger pore sizes, allowing clogging material to penetrate deeper into the specimen, making it more difficult to clean.

#### 3.3.2. Specimens Surface Blockage Morphology

**Figure 18:**
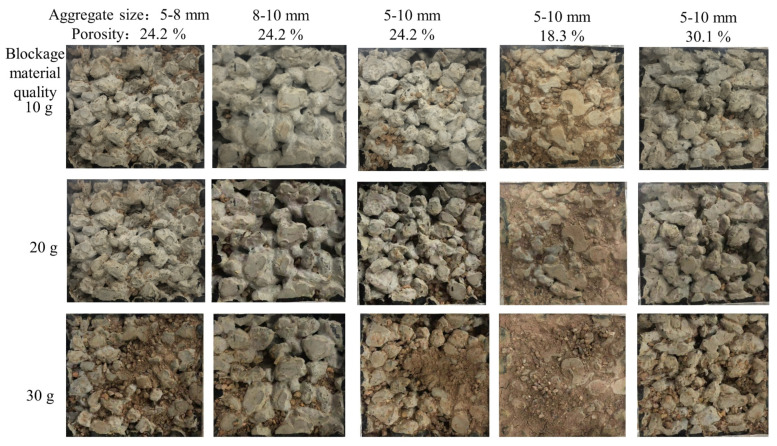
Specimen surface after clogging.

The changes in the pore structure of pervious concrete and the distribution of blockage materials within the pores in three states were studied: unblocked, complete blockage, and blockage cleared. Medical CT scanning was used to obtain CT images of the pervious concrete in the three states. Figure 18 shows the surface morphologies of the specimens with varying levels of blockage materials. It can be observed that specimens with a porosity of 24.2% and aggregate sizes of 5–8 mm, as well as those with a porosity of 18.3% and aggregate sizes of 5–10 mm, exhibited significant blockage. Notably, as the aggregate size and specimen porosity decreased, the blockage materials had more difficulty penetrating the internal pores and tended to accumulate on the specimen’s surface. This indicates that smaller aggregate sizes and lower porosities result in smaller pore diameters in pervious concrete, making it harder for blockage materials to enter the specimen and leading to their accumulation on the surface, which accelerates the development of blockages. A positive aspect is that the blockage materials were more concentrated on the surface, making them easier to remove with high-pressure water jets, thereby resulting in a higher restoration of permeability after maintenance. Conversely, for specimens with larger pore diameters, the blockage materials penetrate deeper into the specimen, making them more challenging to clean and leading to a lower level of permeability restoration.

#### 3.3.3. Blockage Materials Distribution

**Figure 19:**
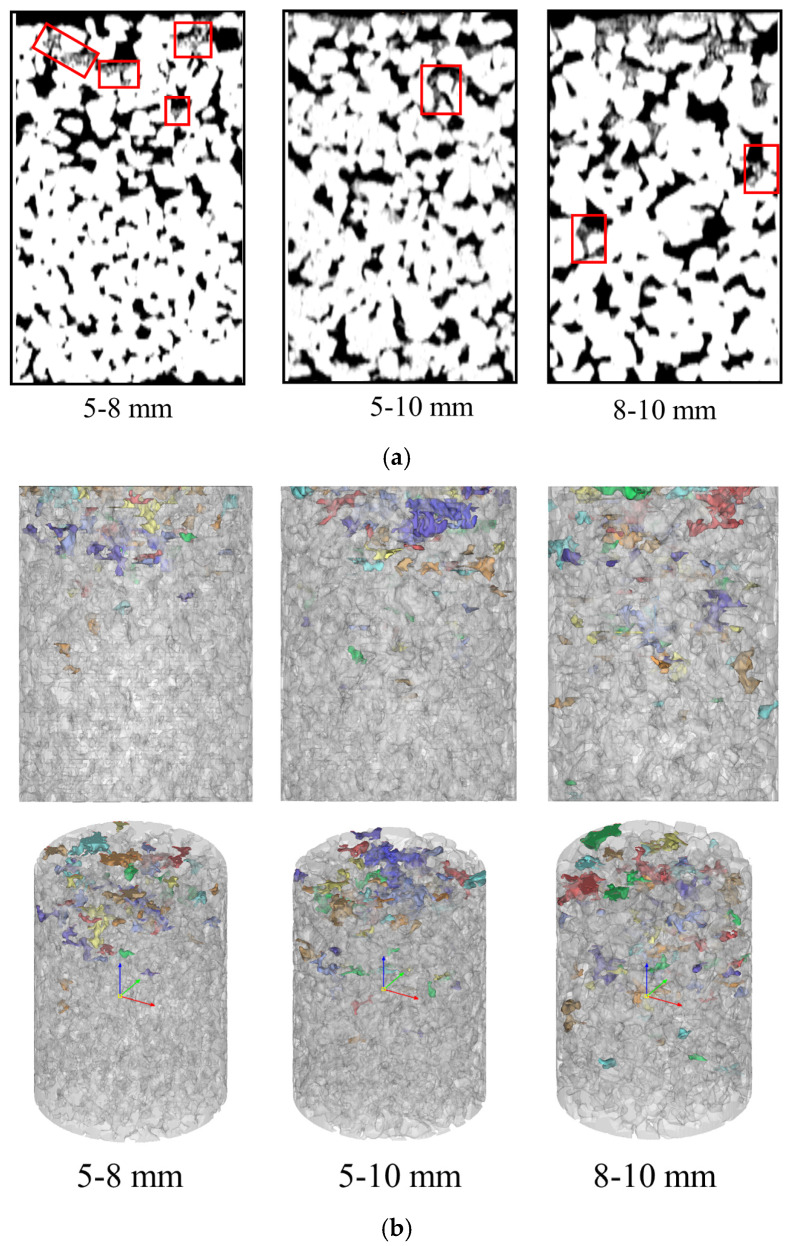
Blockage distribution in specimens with different aggregate sizes: (**a**) 2D distribution; (**b**) 3D distribution.

Figure 19 presents the CT images and three-dimensional distribution of the blockage materials in pervious concrete with different aggregate sizes after complete blockage. In the two-dimensional CT images, the white-highlighted areas represent the pervious concrete, black areas represent pores, and blockage materials located between the black and white regions are indicated by red boxes. As shown in Figure 19, with increasing aggregate size, the blockage materials began to appear deeper within the specimen, and the surface of the specimen became nearly completely blocked. This confirms that larger aggregate sizes with relatively larger pore diameters allow blockage materials to penetrate deeper into the specimen. The advantage of this is that blockage develops more slowly, while the disadvantage is that blockage materials that penetrate deeper into the specimen are more difficult to clean, resulting in a lower level of permeability restoration. In contrast, pervious concrete with smaller aggregate sizes had relatively smaller pore diameters, making it harder for blockage materials to enter the interior of the specimen, leading to their accumulation on the surface. This results in the rapid development of blockage. However, a beneficial aspect was that more blockage materials accumulated on the surface, making them easier to disperse with a high-pressure water jet, resulting in a higher level of permeability restoration after maintenance.

**Figure 20:**
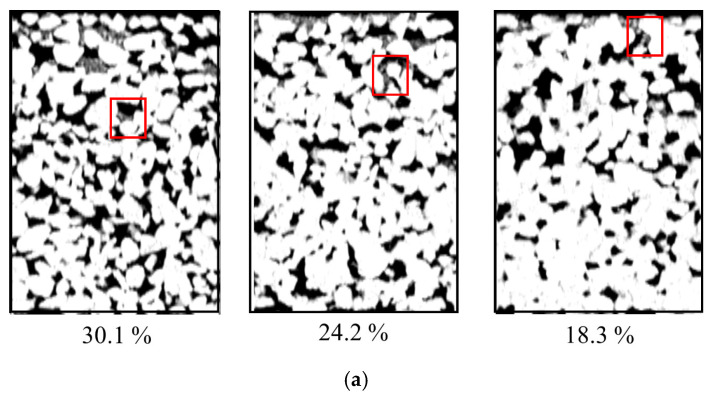

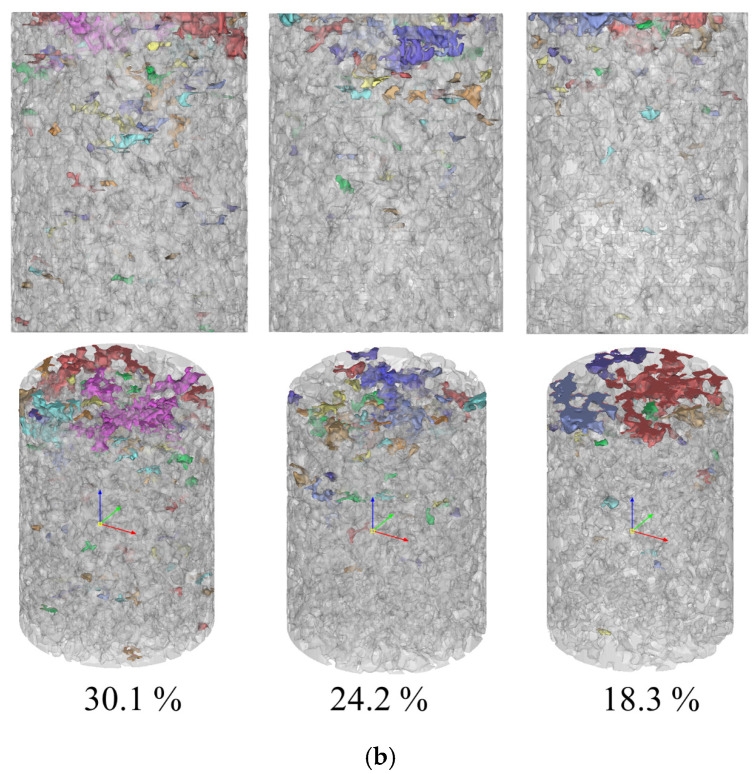
Blockage distribution in specimens with different porosities: (**a**) 2D distribution, (**b**) 3D distribution.

Figure 20 shows the CT images and three-dimensional distribution of the blockage materials in pervious concrete with different porosities after complete blockage. The figure reveals that as the porosity increased, the blockage materials penetrated more easily into the specimen, leading to slower blockage development. However, this also results in blockage materials being more difficult to clean from the interior, resulting in a lower level of permeability restoration.

#### 3.3.4. Variation of Specimens Cross-Sectional Porosity Along the Height

**Figure 21:**
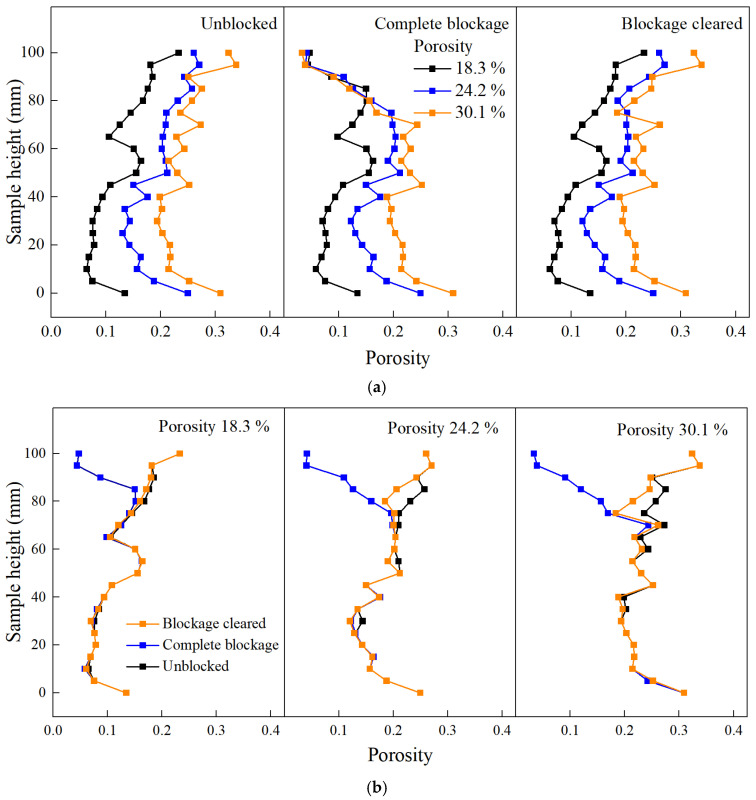
Variation in porosity along height (same aggregate size): (**a**) different porosities; (**b**) different clogging states.

Figure 21 shows the variation in the cross-sectional porosity along the height of the specimens with different porosities in three states: unblocked, completely blocked, and cleaned. As shown in Figure 21a, the cross-sectional porosity along the height of the three pervious concrete specimens showed significant differences before blockage, with the specimen having 30.1% porosity exhibiting greater variation in porosity along its height. After complete blockage, the cross-sectional porosity within the 70–100 mm height range of the specimens tended to become uniform, indicating that the surface layer of the specimen was completely blocked. After cleaning the blockage, the surface blockage materials were removed, and the cross-sectional porosity differences were restored. Figure 21b shows that the blockage was concentrated within the top 30 mm of the surface, and as the specimen porosity increased, the blockage depth gradually increased. After blockage cleaning, the cross-sectional porosity within the upper 0–10 mm range of all specimens was fully restored, indicating that the surface blockage materials were completely removed. However, the cross-sectional porosity within the 10–30 mm range of larger-porosity specimens was not fully restored, and in some cases, it could not be restored at all. This indicates that blockage materials at this depth were not completely removed or were impossible to remove, resulting in a lower level of permeability restoration for larger-porosity specimens.

**Figure 22:**
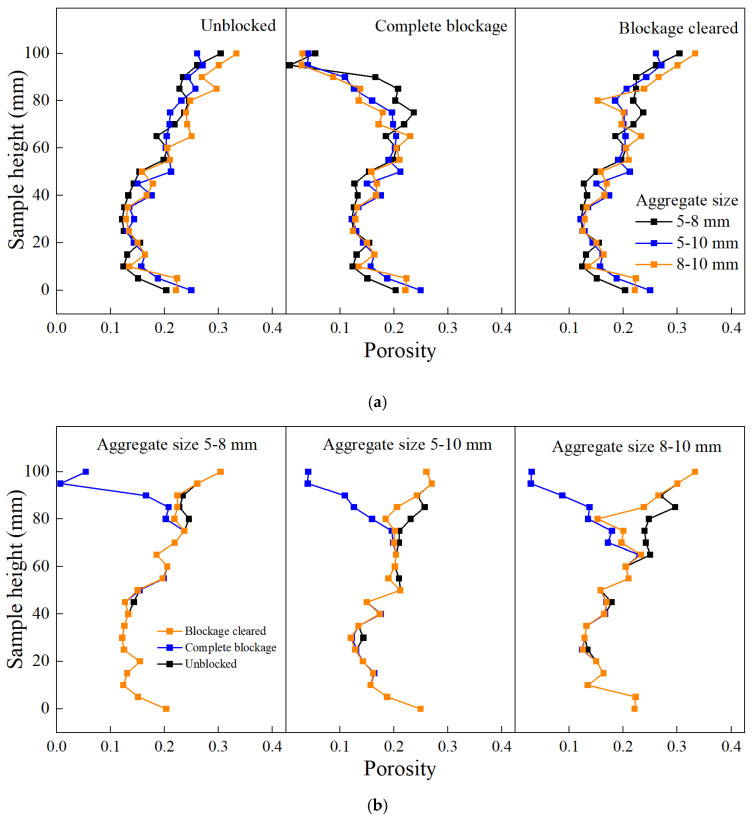
Variation in porosity along the height (specimens with different aggregate sizes): (**a**) different aggregate sizes and (**b**) different clogging states.

Figure 22 shows the variation in the cross-sectional porosity along the height of the specimens with different aggregate sizes in the three states. As shown in Figure 22a, before blockage, the cross-sectional porosity variation along the height of the pervious concrete with the three aggregate sizes was not significantly different. After complete blockage, the cross-sectional porosity within the 0–10 mm surface range of each specimen tended to become uniform, indicating that the blockage materials were mainly concentrated on the specimen surface. Figure 22b reveals that as the aggregate size increased, there was a noticeable decrease in the cross-sectional porosity at greater depths, indicating that the blockage materials gradually penetrated deeper into the specimen. For specimens with aggregate sizes of 5–8 mm and 5–10 mm, the blockage depth ranged from 0–30 mm, while for specimens with aggregate sizes of 8–10 mm, the blockage depth ranged from 0–40 mm, indicating that blockage materials penetrated the interior of these specimens more easily. After the blockage was cleaned, the restoration of cross-sectional porosity within the 0–10 mm surface range was complete for all specimens with different aggregate sizes. For specimens with aggregate sizes of 5–8 mm and 5–10 mm, the cross-sectional porosity within the 10–30 mm range can be partially restored, while for specimens with aggregate sizes of 8–10 mm, the cross-sectional porosity within the 10–40 mm range is difficult to restore. This indicates that as the aggregate size increased, the blockage materials penetrated the interior of the specimen more easily, making them harder to remove and resulting in a lower level of permeability restoration.

#### 3.3.5. Changes in Specimen Pore Size During the Blockage Process

**Figure 23:**
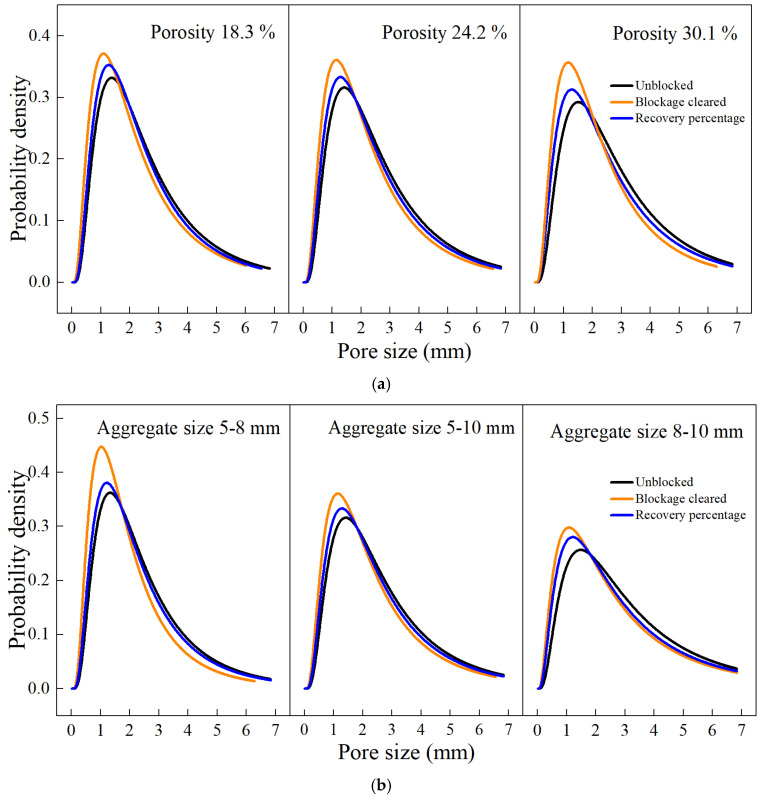
Variation in pore size with different clogging states: (**a**) different porosities; (**b**) different aggregate sizes.

Figure 23 shows the variation in pore size within the top 30 mm of the specimen surface across different blockage states. The pore size distribution follows a lognormal distribution. As observed in the figure, before blockage, the surface pores are relatively large. After complete blockage, larger and more connected pores were obstructed, reducing their proportion. In contrast, smaller pores, due to their poor connectivity and higher tortuosity, were less likely to be blocked, resulting in an increased proportion of small pores. After the blockage was cleaned, the pore size distribution did not revert to the pre-blockage state, primarily because the blockage materials at deeper levels could not be completely removed. Smaller aggregate specimens showed better restoration of porosity in deeper layers (10–30 mm), whereas larger aggregates (8–10 mm) retained deeper blockages (10–40 mm), making full permeability recovery challenging. This indicates that effective cleaning methods need to focus on penetrating deeper blockages, especially in specimens with larger aggregates, to improve long-term permeability restoration.

#### 3.3.6. Permeability Degradation Prediction

By employing a multiple linear regression approach, a degradation model for the permeability coefficient of the aggregate concrete was established. This model correlates the unit area blockage mass, porosity, and the average aggregate particle size (with average values of 6.5 mm, 7.5 mm, and 9 mm for aggregates of 5–8 mm, 5–10 mm, and 8–10 mm, respectively) with the measured permeability coefficient, as shown in Equation (3). According to statistics [40,41], the daily average blockage in coastal city permeable pavements is 0.332 g/m^2^/day. Assuming that no maintenance is performed during the use of the permeable pavement, this value allows the degradation model to be transformed into a service life prediction model for permeability performance, as illustrated in Equation (4).

(4) $$K=-0.00103M+26.32P+1.03Ra-7.98\cdot R^{2}=0.95$$

where *K* represents the permeability coefficient (mm/s), *M* denotes the mass of blockage per unit area (g/m^2^), *P* stands for the porosity of the permeable concrete, and *R*a indicates the average particle size of the aggregate (mm).

(5) $$N=\frac{K_{N}-26.32P-1.03\mathrm{Ra}+7.98}{-0.00103\times 0.332\times 365}$$

where *N* represents the service life of the permeability performance (years), and *K_N_* denotes the minimum permissible permeability coefficient during service (mm/s).

## 4. Conclusions

This study employed CT imaging technology to comprehensively analyze the effects of pore size characteristics and cement paste distribution on the strength and permeability of permeable concrete. Additionally, it considered the impact of blockage consolidation due to dry-wet cycles. From a microstructural perspective, this study revealed the degradation process and maintenance mechanisms of the permeability performance. The main conclusions are as follows:

1. Increased porosity significantly reduced the compressive strength, with values dropping from 25.6 MPa at 18.3% porosity to 17.3 MPa at 30.1%. Permeability increased with higher porosity, from 4.16 mm/s at 18.3% to 7.76 mm/s at 30.1%. Larger aggregate sizes also enhanced permeability, with coefficients ranging from 5.23 mm/s for 5–8 mm aggregates to 6.52 mm/s for 8–10 mm aggregates. However, further increases in aggregate size yield smaller gains in permeability, indicating a balance between porosity, aggregate size, and overall performance.

2. As the porosity increased, the range of pore sizes expanded, and the proportion of the bonding region between the cement paste and aggregates decreased. For permeable concrete with a porosity of 24.2%, the bonding area of the cement paste with aggregates was reduced by 26.9% compared to that of concrete with a porosity of 18.3%. For concrete with a porosity of 30.1%, this reduction reached 36.5%. In contrast to porosity, variations in aggregate particle size have a less significant impact on compressive strength and permeability. As the aggregate particle size increased, the number of pores decreased, while the pore size and the thickness of cement paste adhering to the aggregates increased. For aggregates with a particle size of 8–10 mm, pore sizes are primarily within the range of 2.2–4.4 mm, with some larger pores exceeding 5.5 mm.

3. The distribution of the cement paste thickness within the permeable concrete follows a two-parameter Weibull distribution, with the scale parameter (λ) and shape parameter (k) representing the distribution patterns. This quantification of the cement paste thickness offers valuable insights for understanding and modeling the microstructure of permeable concrete, aiding in the optimization of the mix design to balance strength and permeability.

4. The pore size distribution in permeable concrete can be effectively modeled using a lognormal distribution with parameters that can quantify the permeability performance of the material. Understanding this distribution provides a basis for optimizing the concrete mix to achieve the desired permeability while maintaining structural integrity.

5. Permeable concrete with larger aggregate sizes (8–10 mm) and higher porosity (30.1%) is capable of accommodating more blockages, leading to a slower rate of blockage development. However, post-maintenance permeability recovery is less effective. A detailed quantitative study of the blockage distribution, depth, and pore size changes was conducted across three states: unblocked, completely blocked, and blockage cleared. The findings revealed that blockages were mainly concentrated within the top 0–30 mm of the permeable concrete surface. As the pore size and aggregate particle size increase, blockages tend to penetrate deeper into the concrete, with blockages in the 10–30 mm range being particularly resistant to complete removal, even with high-pressure water jets. A degradation model for the permeability performance of permeable concrete, which accounts for the consolidation of blockages, was established using parameters such as the blockage accumulation per unit area, aggregate particle size, and concrete porosity. This model provides a theoretical and data-driven framework for assessing the service life of permeable concrete under different blockage conditions.

This study provides valuable insights into optimizing permeable concrete mix designs by balancing porosity, aggregate size, and strength for better performance in urban infrastructure. Additionally, research on blockage consolidation and permeability degradation offers guidance for effective maintenance practices, particularly for removing deep blockages that are resistant to high-pressure water jets. Furthermore, the developed permeability degradation model helps predict the service life of permeable concrete, enabling more sustainable and cost-effective infrastructure planning.

This study has several limitations that should be acknowledged. Firstly, the research primarily focused on the effects of porosity and aggregate size on strength and permeability, but did not extensively explore the influence of other factors such as cement type, water-cement ratio, or curing conditions, which could also significantly impact the performance of permeable concrete. Secondly, while the study provided a detailed analysis of blockage consolidation and permeability degradation, the experiments were conducted under controlled laboratory conditions, which may not fully replicate the complex and variable environmental conditions encountered in real-world applications. Additionally, the long-term effects of freeze-thaw cycles and the interaction of different types of pollutants with the pore structure were not investigated, which are critical for understanding the durability of permeable concrete in diverse climates. Future research should address these limitations to provide a more comprehensive understanding of permeable concrete performance and develop more robust and sustainable urban infrastructure solutions.

Future research should focus on further optimizing the mix design of permeable concrete, incorporating alternative aggregate materials, and exploring the impact of various environmental factors on permeability and strength over time. Additionally, advancements in monitoring and assessing the long-term effects of freeze-thaw cycles and the development of advanced maintenance technologies for more efficient blockage removal are areas of interest. We also plan to investigate the impact of different types of pollutants and their interactions with pore structures on the degradation of permeable concrete. These areas will contribute to a better understanding of the durability and sustainability of permeable concrete in real-world applications.

## Figures and Tables

**Table 1:** Different aperture feature analysis methods on a two-dimensional scale.

Reference	Water-to-Binder Ratio	Aggregate Size (mm)	Pore Characterization Method
[13]	0.3	4.75–9.5	Slicing-shooting—gray value processing
[14]	0.3	2.36–4.75 4.75–9.5 9.5–12.5	Slicing-scanning—gray value processing
[15]	0.25, 0.3, 0.35, 0.4, 0.45, 0.5	3.75, 7.5, 13	beetle antennae search (BAS)
[16]	0.26	5–10, 10–15	Slicing-shooting—gray value processing
[17]	0.28, 0.30, 0.32, 0.35	4.75–9.5	Katz-Thompson model (K-T)

**Table 2:** Cement compositions.

Composition	CaO	SiO_2_	Al_2_O_3_	Fe_2_O_3_	MgO	SO_3_	K_2_O	Na_2_O
Percentage (%)	64.68	21.58	4.27	3.42	2.11	2.45	0.56	0.10

**Table 3:** Physical and mechanical properties of Ordinary Portland Cement.

Density (kg/m^3^)	Specific Surface Area (m^2^/kg)	Setting Time (min)		Compressive Strength (MPa)		Flexural Strength (MPa)	
		Initial Set	Final Set	3 d	28 d	3 d	28 d
3110	340	175	245	27.5	48.2	5.5	7.4

**Table 4:** Physical and mechanical properties of the aggregates.

Apparent Density (kg/m^3^)	Compact Bulk Density (kg/m^3^)	Water Absorption (%)	Crushing Index (%)
2840	1570	0.6	11.8

**Table 5:** Mix proportions.

Sample	Aggregate Size (mm)	Compact Bulk Porosity (%)	Aggregate Mass (kg/m^3^)	Cement Mass (kg/m^3^)	Water-Cement Ratio	Porosity (%)
PC1	5–8	45.1	1270	385	0.3	24.2
PC2	8–10	44.3	1270	374	0.3	24.2
PC3	5–10	44.7	1270	285	0.3	30.1
PC4	5–10	44.7	1270	380	0.3	24.2
PC5	5–10	44.7	1270	475	0.3	18.3

**Table 6:** *µ* and *σ* of Lognormal distribution.

Variable	*µ*	*σ*
Porosity 30.1%	2.62	1.90
Porosity 24.2%	2.39	1.64
Porosity 18.3%	2.48	1.69
Aggregate size 5–8 mm	2.22	1.45
Aggregate size 5–10 mm	2.87	1.81
Aggregate size 8–10 mm	2.48	1.69

**Table 7:** *λ* and *k* of Lognormal distribution.

Variable	*λ*	*k*
Porosity 30.1%	0.51	2.16
Porosity 24.2%	0.71	2.15
Porosity 18.3%	0.92	1.91
Aggregate size 5–8 mm	0.86	1.99
Aggregate size 5–10 mm	1.10	2.09
Aggregate size 8–10 mm	0.92	1.91

**Table 8:** The comparative fit of the Weibull and lognormal distributions is shown in Figure 13a.

Weibull distribution of Figure 13a					
a		b		statistics	
Value	Standard Erro	Value	Standard Erro	Reduced Chi-Sqr	Adj. R-Square
0.53604	0.02182	0.33676	0.03045	0.00709	0.97198
0.78801	0.07604	0.52726	0.10753	0.01104	0.94737
0.96394	0.13946	0.67564	0.20029	0.01172	0.81613
lognormal distribution of Figure 13a					
a		b		statistics	
Value	Standard Erro	Value	Value	Standard Erro	Value
0.50873	0.01004	2.15659	0.07001	0.01214	0.96913
0.71359	0.0168	2.14691	0.08257	0.01217	0.94196
0.92005	0.03422	1.91362	0.10239	0.01708	0.81409

## Data Availability

The original contributions presented in the study are included in the article, further inquiries can be directed to the corresponding author.

## References

[1] Jiménez J.R., Ayuso J., Agrela F., López M., Galvín A.P. (2012). Utilisation of Unbound Recycled Aggregates from Selected CDW in Unpaved Rural Roads. Resour. Conserv. Recycl..

[2] Nantasai B., Nassiri S. (2019). Winter Temperature Prediction for Near-Surface Depth of Pervious Concrete Pavement. Int. J. Pavement Eng..

[3] Xie X., Zhang T., Wang C., Yang Y., Bogush A., Khayrulina E., Huang Z., Wei J., Yu Q. (2020). Mixture Proportion Design of Pervious Concrete Based on the Relationships between Fundamental Properties and Skeleton Structures. Cem. Concr. Compos..

[4] Kant Sahdeo S., Ransinchung G.D., Rahul K.L., Debbarma S. (2020). Effect of Mix Proportion on the Structural and Functional Properties of Pervious Concrete Paving Mixtures. Constr. Build. Mater..

[5] Claudino G.O., Rodrigues G.G.O., Rohden A.B., Mesquita E.F.T., Garcez M.R. (2022). Mix Design for Pervious Concrete Based on the Optimization of Cement Paste and Granular Skeleton to Balance Mechanical Strength and Permeability. Constr. Build. Mater..

[6] Poon C.S., Lam C.S. (2008). The Effect of Aggregate-to-Cement Ratio and Types of Aggregates on the Properties of Pre-Cast Concrete Blocks. Cem. Concr. Compos..

[7] Liu H., Luo G., Wei H., Yu H. (2018). Strength, Permeability, and Freeze-Thaw Durability of Pervious Concrete with Different Aggregate Sizes, Porosities, and Water-Binder Ratios. Appl. Sci..

[8] Tang C.W., Cheng C.K., Tsai C.Y. (2019). Mix Design and Mechanical Properties of High-Performance Pervious Concrete. Materials.

[9] Wu S., Wu Q., Shan J., Cai X., Su X., Sun X. (2023). Effect of Morphological Characteristics of Aggregate on the Performance of Pervious Concrete. Constr. Build. Mater..

[10] Chockalingam T., Vijayaprabha C., Leon Raj J. (2023). Experimental Study on Size of Aggregates, Size and Shape of Specimens on Strength Characteristics of Pervious Concrete. Constr. Build. Mater..

[11] Ćosić K., Korat L., Ducman V., Netinger I. (2015). Influence of Aggregate Type and Size on Properties of Pervious Concrete. Constr. Build. Mater..

[12] Zhang Z., Zhang Y., Yan C., Liu Y. (2017). Influence of Crushing Index on Properties of Recycled Aggregates Pervious Concrete. Constr. Build. Mater..

[13] Liao L., Wu S., Hao R., Zhou Y., Xie P. (2023). The Compressive Strength and Damage Mechanisms of Pervious Concrete Based on 2D Mesoscale Pore Characteristics. Constr. Build. Mater..

[14] Neithalath N., Sumanasooriya M.S., Deo O. (2010). Characterizing Pore Volume, Sizes, and Connectivity in Pervious Concretes for Permeability Prediction. Mater. Charact..

[15] Sun J., Zhang J., Gu Y., Huang Y., Sun Y., Ma G. (2019). Prediction of Permeability and Unconfined Compressive Strength of Pervious Concrete Using Evolved Support Vector Regression. Constr. Build. Mater..

[16] Liu R., Chi Y., Chen S., Jiang Q., Meng X., Wu K., Li S. (2020). Influence of Pore Structure Characteristics on the Mechanical and Durability Behavior of Pervious Concrete Material Based on Image Analysis. Int. J. Concr. Struct. Mater..

[17] Debnath B., Sarkar P.P. (2019). Permeability Prediction and Pore Structure Feature of Pervious Concrete Using Brick as Aggregate. Constr. Build. Mater..

[18] Zhong R., Wille K. (2016). Linking Pore System Characteristics to the Compressive Behavior of Pervious Concrete. Cem. Concr. Compos..

[19] Shan J., Zhang Y., Wu S., Lin Z., Li L., Wu Q. (2022). Pore Characteristics of Pervious Concrete and Their Influence on Permeability Attributes. Constr. Build. Mater..

[20] Huang J., Zhang Y., Sun Y., Ren J., Zhao Z., Zhang J. (2021). Evaluation of Pore Size Distribution and Permeability Reduction Behavior in Pervious Concrete. Constr. Build. Mater..

[21] Deo O., Neithalath N. (2010). Compressive Behavior of Pervious Concretes and a Quantification of the Influence of Random Pore Structure Features. Mater. Sci. Eng. A.

[22] Cai J., Chen J., Shi J., Tian Q., Xu G., Du Y. (2022). A Novel Approach to Evaluate the Clogging Resistance of Pervious Concrete. Case Stud. Constr. Mater..

[23] Nan X., Wang Z., Hou J., Tong Y., Li B. (2021). Clogging Mechanism of Pervious Concrete: From Experiments to CFD-DEM Simulations. Constr. Build. Mater..

[24] Sandoval G.F.B., Pieralisi R., Dall Bello de Souza Risson K., Campos de Moura A., Toralles B.M. (2022). Clogging Phenomenon in Pervious Concrete (PC): A Systematic Literature Review. J. Clean. Prod..

[25] Cui X., Zhang X., Wang J., Zhang J., Qi H., Li J. (2020). X-Ray CT Based Clogging Analyses of Pervious Concrete Pile by Vibrating-Sinking Tube Method. Constr. Build. Mater..

[26] Kayhanian M., Anderson D., Harvey J.T., Jones D., Muhunthan B. (2012). Permeability Measurement and Scan Imaging to Assess Clogging of Pervious Concrete Pavements in Parking Lots. J. Environ. Manag..

[27] Kia A., Wong H.S., Cheeseman C.R. (2017). Clogging in Permeable Concrete: A Review. J. Environ. Manag..

[28] Deo O., Sumanasooriya M., Neithalath N. (2010). Permeability Reduction in Pervious Concretes due to Clogging: Experiments and Modeling. J. Mater. Civ. Eng..

[29] Sandoval G.F.B., Galobardes I., De Moura A.C., Toralles B.M. (2020). Hydraulic Behavior Variation of Pervious Concrete due to Clogging. Case Stud. Constr. Mater..

[30] Tan S.-A., Fwa T.-F., Han C.-T. (2003). Clogging Evaluation of Permeable Bases. J. Transp. Eng..

[31] Winston R.J., Al-Rubaei A.M., Blecken G.T., Viklander M., Hunt W.F. (2016). Maintenance Measures for Preservation and Recovery of Permeable Pavement Surface Infiltration Rate—The Effects of Street Sweeping, Vacuum Cleaning, High Pressure Washing, and Milling. J. Environ. Manag..

[32] Yuan J., Chen X., Liu S., Li S., Shen N. (2018). Effect of Water Head, Gradation of Clogging Agent, and Horizontal Flow Velocity on the Clogging Characteristics of Pervious Concrete. J. Mater. Civ. Eng..

[33] Wang X., Wang Y., Ge X., Tong B., Schaefer V., Wang K., Li C. (2022). The Quantitative Assessment of Clogging and Cleaning Effects on the Permeability of Pervious Concrete. Constr. Build. Mater..

[34] Lee J.-W., Yang E., Jang J., Yun T.S. (2022). Effect of Clogging and Cleaning on the Permeability of Pervious Block Pavements. Int. J. Pavement Eng..

[35] Brugin M., Marchioni M., Becciu G., Giustozzi F., Toraldo E., Andrés-Valeri V.C. (2020). Clogging Potential Evaluation of Porous Mixture Surfaces Used in Permeable Pavement Systems. Eur. J. Environ. Civ. Eng..

[36] Wang Z., Zou D., Liu T., Zhou A., Shen M. (2020). A Novel Method to Predict the Mesostructure and Performance of Pervious Concrete. Constr. Build. Mater..

[37] Deb P., Debnath B., Hasan M., Alqarni A.S., Alaskar A., Alsabhan A.H., Khan M.A., Alam S., Hashim K.S. (2022). Development of Eco-Friendly Concrete Mix Using Recycled Aggregates: Structural Performance and Pore Feature Study Using Image Analysis. Materials.

[38] Wang Z., Zou D., Liu T., Zhou A. (2021). Influence of Paste Coating Thickness on the Compressive Strength, Permeability, and Mesostructure of Permeable Concrete. Constr. Build. Mater..

[39] Barišić I., Grubeša I.N., Dokšanović T., Zvonarić M. (2020). Influence of Clogging and Unbound Base Layer Properties on Pervious Concrete Drainage Characteristics. Materials.

[40] Zhou H., Li H., Abdelhady A., Liang X., Wang H., Yang B. (2019). Experimental Investigation on the Effect of Pore Characteristics on Clogging Risk of Pervious Concrete Based on CT Scanning. Constr. Build. Mater..

[41] Lin W., Park D.-G., Ryu S.W., Lee B.-T., Cho Y.-H. (2016). Development of Permeability Test Method for Porous Concrete Block Pavement Materials Considering Clogging. Constr. Build. Mater..

